# Cardiovascular risk and obesity in miner workers exposed to intermittent hypobaric hypoxia in the Peruvian Andes

**DOI:** 10.3389/fphys.2026.1693470

**Published:** 2026-03-10

**Authors:** Elizbet S. Montes-Madariaga, Brando Ortiz-Saavedra, Julio S. Mamani-Castillo, Anel Ivonne Valencia-Pacheco, Brenda Oporto-Arenas, Jose Luis Manrique-Ccopa, Jorge Ballón-Echegaray, Irmia Paz, Ricardo David Davila Ralaiza, Arturo A. Arce-Esquivel, Oscar Moreno-Loaiza, Ricardo A. J. Leon-Vasquez

**Affiliations:** 1 Faculty of Medicine, Universidad Nacional de San Agustín de Arequipa, Arequipa, Peru; 2 Biology Study Program, Faculty of Biological Sciences, Universidad Nacional de San Agustín de Arequipa, Arequipa, Peru; 3 Department of Nutritional Sciences, Faculty of Biological Sciences, Universidad Nacional de San Agustín de Arequipa, Arequipa, Peru; 4 Department of Kinesiology, School of Health Professions, The University of Texas at Tyler, Tyler, TX, United States; 5 Institute of Biophysics Carlos Chagas Filho, Federal University of Rio de Janeiro, Rio de Janeiro, Brazil

**Keywords:** altitude, cardiovascular risk, hypobaric hypoxia, hypoxia, obesity risk

## Abstract

**Background:**

Occupational activities such as mining and interprovincial transportation expose Peruvian workers to large altitude fluctuations, generating repeated cycles of chronic intermittent hypobaric hypoxia (CIHH). In this study, we aimed to evaluate the effect of CIHH exposure on cardiovascular risk, obesity, sleep quality, and physical activity among male workers performing rotational shifts at high altitude.

**Methods:**

A cross-sectional study was conducted with 96 male participants aged 20–60 years, who were divided into two groups: those exposed to CIHH (n = 53; ≥3,000 m, alternating with <2,500 m) and controls working permanently at low altitude (<2,500 m; n = 43). Anthropometric and body composition parameters were assessed by bioelectrical impedance, and cardiovascular risk was estimated using the Reynolds Risk Score (RRS) and Framingham Risk Score (FRS). Sleep apnea risk and physical activity were evaluated using the STOP-Bang Questionnaire and the International Physical Activity Questionnaire—Short Form (IPAQ-SF), respectively. Biochemical and hematological profiles were also analyzed.

**Results:**

Obesity prevalence was significantly higher in the CIHH group (80%) than in controls (20%) (p = 0.036; PR = 1.58, 95% CI: 1.12–2.24). Despite this, body fat (56.8% vs. 43.2%, p = 0.5), skeletal muscle (55.2% vs. 44.8%, p = 0.9), and visceral fat (57.8% vs. 51.0%, p = 0.5) did not differ significantly. Hematocrit levels were higher in CIHH workers (median 52.3%) than in controls (50.7%) (p = 0.03), indicating mild erythropoietic adaptation. No significant between-group differences were observed in lipid profile, glucose, insulin, high-sensitivity C-reactive protein (hs-CRP), interleukin-6 (IL-6), physical activity, or obstructive sleep apnea (OSA) risk (all p > 0.05). Estimated cardiovascular risk remained low and comparable between groups.

**Conclusion:**

CIHH exposure was associated with higher hematocrit levels and a significantly greater prevalence of obesity but no adverse alterations in cardiovascular or metabolic biomarkers. These findings suggest a dual physiological response to intermittent hypoxia—erythropoietic adaptation coexisting with metabolic vulnerability—highlighting the need for preventive strategies in high-altitude occupational health.

## 1 Introduction

Roughly one-third of Peru’s population resides above 2,500 m above sea level (masl) (INEI, 2023). The Andean geography creates a unique environment where many individuals routinely commute between lowland and high-altitude regions for occupational purposes. Over the last decade, large-scale mining and long-distance transport have intensified this vertical mobility, exposing workers to alternating periods of low and high barometric and oxygen pressures (Ric et al., 2004). Such cyclical exposure defines a physiological and occupational model known as chronic intermittent hypobaric hypoxia (CIHH) (Farías et al., 2013; Viscor et al., 2018; Aragón-Vela et al., 2020).

CIHH refers to repeated exposure to low-oxygen and low-pressure conditions typically found at altitudes above 3,000 m, interspersed with recovery periods at lower elevations (Moore, 2004; Müller et al., 2025; González-Candia et al., 2023). From a physiological standpoint, exposure exceeding 2,500 m is already sufficient to trigger measurable hematological and metabolic adaptations (Brito et al., 2018). According to the Chilean Technical Guide on Occupational Hypobaria (Ministerio de salud de Chile, 2022), high altitude is defined as 3,000–5,500 m, and a worker is considered chronically and intermittently exposed when at least 30% of total working time is spent at high altitude for 6 months or longer.

The hypoxia–reoxygenation cycles characteristic of CIHH activate molecular pathways such as HIF-1α, NF-κB, and eNOS, promoting oxidative stress, endothelial dysfunction, and systemic inflammation (Yuan et al., 2017; Ren et al., 2024; Calderon-Jofre et al., 2022; Cui et al., 2020). Although these responses may contribute to acclimatization by improving oxygen transport and erythropoiesis (Mallet et al., 2021), chronic exposure has also been linked to adverse effects, including vascular remodeling, metabolic dysregulation, and increased cardiovascular risk (Mallet et al., 2021; Hernández-Vásquez et al., 2022; Lüneburg et al., 2017; Pedreros-Lobos et al., 2021).

Globally, cardiovascular diseases (CVDs) remain the leading cause of death, accounting for nearly one-third of all mortalities (World Health Organization, 2023). Hypoxia is now recognized as a powerful environmental determinant of cardiometabolic health. The interplay between CIHH, obesity, and obstructive sleep apnea (OSA) could amplify oxidative and inflammatory damage, thereby enhancing cardiovascular risk (Brito et al., 2018; Pedreros-Lobos et al., 2021; Turnbull, 2018; Drager et al., 2013; Bloch et al., 2015).

Despite extensive high-altitude mining and transport activities in the Andes, few studies have explored these interactions in Peruvian workers, and most lacked low-altitude control groups (Hernández-Vásquez et al., 2022; Woolcott et al., 2016). Moreover, physiological adaptations unique to Andean populations may alter the impact of hypobaric exposure on metabolic and cardiovascular outcomes.

Therefore, in this study, we aimed to evaluate the association between CIHH exposure, obesity, and cardiovascular risk among workers performing rotational high-altitude shifts in Peru. Understanding these interactions is essential to guide preventive and monitoring strategies in occupational health within high-altitude environments.

## 2 Methods

### 2.1 Ethical consideration

The research protocol was reviewed and approved by the Institutional Research Ethics Committee (CIEI) of the Universidad Peruana Cayetano Heredia (UPCH) under registration code 21005 and certificate number R-028-01-23.

All participants provided written informed consent prior to enrolment. The study adhered to the Declaration of Helsinki (2013 revision) (World Medical Association, 2013) and complied with national Peruvian standards for biomedical research involving human subjects. Data confidentiality was ensured through coded identification and restricted access to all databases. Only authorized investigators could access participant information, which was stored securely and anonymized for analysis.

### 2.2 Study design and participants

This was an observational, cross-sectional study conducted in Arequipa, Peru (2,335 m a.s.l.). Two groups were defined according to occupational exposure:

- The CIHH group, composed of workers performing rotational shifts at altitudes ≥3,000 m, alternating with rest periods at <2,500 m.
- The control group, consisting of workers with permanent work schedules at low altitude (<2,500 m).

To maintain sample homogeneity, only male participants aged 20–60 years were included. Female workers were excluded to avoid potential hormonal and inflammatory variability associated with menstrual cycles and reproductive physiology (De Souza et al., 2005).

A total of 100 volunteers were initially enrolled. After applying inclusion and exclusion criteria, 96 participants were retained for final analysis—53 (55.2%) exposed and 43 (44.8%) controls. The sample size was calculated to compare means between two groups using a two-tailed hypothesis test, a 99% confidence level, and 80% statistical power, based on body mass index (BMI) variance (2.6) from the study by Pizarro-Montaner et al. (2020). The detectable difference (d) was set at 1 (d = 1). After adjusting for a 20% potential loss, a total of 97 participants were required.

### 2.3 Inclusion and exclusion criteria

#### 2.3.1 Inclusion criteria

- Male workers aged 20–60 years.
- Exposure to rotational shifts at ≥3,000 m, alternating with rest periods at <2,500 m for at least six consecutive months (CIHH group).
- Permanent work at <2,500 m (control group).

#### 2.3.2 Exclusion criteria

- BMI <18.5 or >40 kg/m^2^.
- Previous diagnosis of hypertension, coronary heart disease, diabetes mellitus, cancer, COPD, or chronic inflammatory conditions prior to occupational activity.
- Acute illness, antibiotic use, or anti-inflammatory use within the last 30 days.
- Chronic mountain sickness diagnosed before enrolment (León-Velarde et al., 2005).
- Smoking ≥1 cigarette per day or having quit within the previous year.

### 2.4 Exposure assessment

Exposure to CIHH was defined as performing rotational work at altitudes ≥3,000 m, alternating with rest periods at <2,500 m (Pollard and Murdoch, 2003; Savioli et al., 2022). The mean workplace altitude among exposed workers was 4,050 ± 400 m, with a range of 3,000–>4,500 m.

Rotational schedules ranged from 4 × 3 to 21 × 7 days, with an average of 9.9 ± 4.6 workdays followed by 6.2 ± 3.1 rest days. Altitude exposure was verified through official employment records provided by each worker’s company, indicating workplace location and altitude (e.g., “Arequipa–Puno” or “Arequipa–Mollendo”). Control group participants worked continuously at low altitude (<2,500 m).

### 2.5 Body composition and anthropometric evaluation

Anthropometric and body composition measurements were performed in the morning after an overnight fast, concurrently with fasting blood sampling. Participants were measured barefoot, wearing light clothing, and without accessories. Waist circumference was measured directly over the skin following standard anatomical landmarks (Lohman et al., 1988).

Measurements were performed using the Omron HBF-514C (Omron Healthcare, Japan), providing bioelectrical impedance analysis for total fat, visceral fat, and skeletal muscle mass. Height was recorded using a calibrated stadiometer, and body weight was measured using a digital scale. BMI was calculated as weight (kg)/height^2^ (m^2^) and classified according to WHO standards (World Health Organization, 2000). Abdominal obesity was defined as waist ≥94 cm (ALAD criteria) (Asociación Latinoamericana de Diabetes, 2019), and the conicity index was computed following the study by Valdez (1991). All equipment was calibrated and certified by the UNSA laboratory.

### 2.6 Biochemical and cardiovascular parameters

Venous blood samples (10 mL) were obtained from the antecubital vein after fasting. Samples were centrifuged and processed immediately in UNSA’s biochemistry and hematology laboratories.

Biochemical parameters—glucose, total cholesterol, HDL-C, LDL-C, and triglycerides—were determined using semi-automated spectrophotometry (BA-88A, Mindray, China) according to Valtek Diagnostics (Chile) protocols. Insulin was measured using ELISA, and inflammatory markers [high-sensitivity C-reactive protein (hs-CRP) and interleukin-6 (IL-6)] were quantified with commercial ELISA kits validated for research use.

Hematological parameters (Hb, Hct, total WBC, neutrophils, and lymphocytes) were analyzed using a Zybio Z3 CRP analyzer (Zybio, China). Cardiovascular risk was calculated using both the Reynolds Risk Score (RRS) and Framingham Risk Score (FRS) (D’Agostino et al., 2008), integrating age, sex, systolic blood pressure, antihypertensive therapy, smoking, diabetes, HDL-C, total cholesterol, and hs-CRP.

### 2.7 Questionnaires and clinical variables

Lifestyle and clinical data were collected using a standardized data collection form developed for this study, recording demographics, alcohol and tobacco use, medical history, and work regimen (rotation days, years of exposure, and altitude).

The STOP-Bang Questionnaire (Chung et al., 2016) assessed OSA risk (low, intermediate, or high). The International Physical Activity Questionnaire—Short Form (IPAQ-SF) (Craig et al., 2003) assessed physical activity (low, moderate, or high).

Smoking and alcohol consumption were categorized as current, former, or never, and former users reported frequency, duration, and cessation period.

Chronic mountain sickness (CMS) was evaluated using the Qinghai diagnostic score (Villafuerte and Corante, 2016), which integrates clinical symptoms (cyanosis, palpitations, headache, *etc*.) and hemoglobin levels. CMS status was included as a confounding variable in the multivariate model.

### 2.8 Statistical analysis

Data were entered into Microsoft Excel and analyzed using R and RStudio (The R Project for Statistical Computing, CRAN, United States). Data normality was verified using the Kolmogorov–Smirnov test. Continuous variables were summarized as mean ± SD or median (IQR), and categorical variables were summarized as n (%).

Between-group comparisons were performed using Student’s t-test, Mann–Whitney U, or Chi-square tests. Associations between CIHH exposure and clinical outcomes were determined using Poisson regression with robust variance, reporting prevalence ratios (PRs) and 95% confidence intervals (CIs). The model was adjusted for age, BMI, exposure duration, physical activity, smoking, alcohol, and CMS.

A p-value <0.05 was considered statistically significant. Data visualization was performed using ggplot2 and tidyverse packages in R.

## 3 Results

A total of 100 male participants were initially screened, of whom 96 fulfilled the inclusion criteria and were included in the final analysis. Fifty-three workers formed the CIHH group, and 43 were assigned to the control group. The median age of participants was 34 years, and approximately three-quarters (74%) were born at low altitude (<2,500 m) (Table 1). Among CIHH workers, the median duration of exposure to high-altitude shifts was 1 year, following work–rest rotations that typically ranged from 10–10 to 20–10 days at elevations between 3,000 and 4,500 m above sea level. No significant differences were observed in baseline demographic or occupational variables between the two groups.

**TABLE 1:** Participants’ characteristics.

Variable	N (%)
Age (years)*	34.0 (27.0–39.0)
Birth city altitude (masl)	
<2,500 (%)	71 (74.0)
2,500–3,500 (%)	9 (9.4)
>3,500 (%)	16 (16.7)
Group (n)	
Control	43 (44.8)
CIHH	53 (55.2)

Masl, meters above sea level; *median (IQR).

**FIGURE 1:**
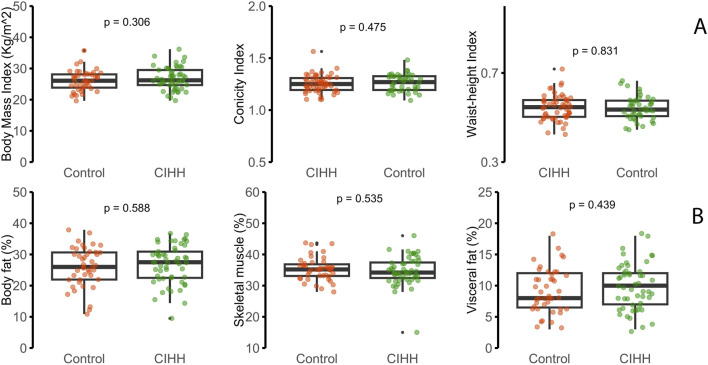
**(A)** Anthropometric measurements (BMI, conicity index, and waist-height index). **(B)** Body composition parameters [body fat (%), skeletal muscle (%), and visceral fat (%)]. Data are presented as box plots, where the central line represents the median, the box limits represent the first and third quartiles, and whiskers extend from the minimum to the maximum. P-values were calculated using Mann–Whitney’s test).

Anthropometric and body composition data are summarized in Table 2 and Figure 1. Overall, there were no statistically significant differences between groups in BMI, conicity index, waist-to-height ratio, or body composition parameters such as total body fat, skeletal muscle mass, and visceral fat (p > 0.05 for all comparisons). Nevertheless, the CIHH group showed a notably greater proportion of participants with obesity (80%) than the control group (20%), a difference that reached statistical significance (p = 0.036). In the Poisson regression model, the prevalence of obesity was 58% higher among CIHH workers than in controls (PR = 1.58; 95% CI: 1.12–2.24). No additional differences were detected for the remaining anthropometric variables.

**TABLE 2:** Body composition of CON and CIHH participants.

Variable	Control (N = 43), n (%)	CIHH (N = 53), n (%)	*p*-value	PR (95% CI)	*p*-value
Abdominal obesity					
No	27 (50.0)	27 (50.0)	0.2	Ref.	
Yes	16 (38.1)	26 (61.9)		1.24 (0.86–1.78)	0.25
Obesity (BMI ≥30)					
No	40 (49.4)	41 (50.6)	0.036*	Ref.	
Yes	3 (20.0)	12 (80.0)		1.58 (1.12–2.24)	0.01
Body fat					
Normal	8 (53.3)	7 (46.7)	0.5	Ref.	
High/very high	35 (43.2)	46 (56.8)		1.22 (0.66–2.24)	0.53
Skeletal muscle (n = 95)					
Low	13 (44.8)	16 (55.2)	0.9	Ref.	
Normal/high	30 (44.8)	37 (55.2)		1.00 (0.67–1.50)	0.9
Visceral fat (n = 94)					
Normal	24 (49.0)	25 (51.0)	0.5	Ref.	
High/very high	19 (42.2)	26 (57.8)		1.13 (0.78–1.65)	0.52

CIHH, chronic intermittent hypobaric hypoxia; PR, prevalence ratio; 95% CI, 95% confidence interval. P-values were calculated using the Chi-square test.

In sensitivity analyses (Supplementary Figures S1–S4), continuous adiposity indices—including BMI as a continuous variable, body fat percentage, and waist-to-height ratio—showed largely overlapping distributions between CIHH workers and controls. In contrast, a between-group difference was mainly observed when BMI was dichotomized using standard cutoffs.

Because obesity and sleep-related breathing disorders are often intertwined in hypoxic environments, we further evaluated the risk of OSA and levels of physical activity. According to the STOP-Bang Questionnaire, there were no significant differences in OSA risk between groups (Table 3). Likewise, results from the IPAQ indicated that physical activity levels were comparable across groups, suggesting that the higher obesity rate among CIHH workers could not be attributed to differences in daily activity patterns.

**TABLE 3:** Obstructive sleep apnea risk and physical activity levels of CON and CIHH participants.

Variable	Control (N = 43), n (%)	CIHH (N = 53), n (%)	p-value*	PR (95% CI)	p-value**
STOP-Bang (risk for OSA)					
Low	31 (43.7)	40 (56.3)	0.7	Ref.	
Intermediate/high	12 (48.0)	13 (52.0)		0.92 (0.59–1.44)	0.72
IPAQ					
Low	16 (50.0)	16 (50.0)	0.5	Ref.	
Moderate	27 (42.2)	37 (57.8)		1.16 (0.76–1.75)	0.5

CIHH, chronic intermittent hypobaric hypoxia; PR, prevalence ratio; 95% CI, 95% confidence interval; BMI, body mass index; OSA, obstructive sleep apnea; IPAQ, International Physical Activity Questionnaire (Bloch et al., 2015). P-values were calculated using the Chi-square test * and Poisson regression model with robust variances**.

**FIGURE 2:**
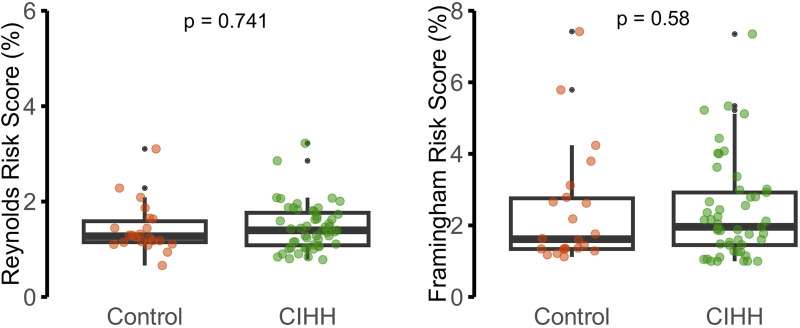
Metabolic parameters: cholesterol (mg/dL), HDL-C (mg/dL), LDL-C (mg/dL), triglycerides (mg/dL), glucose (mg/dL), and insulin (mIU/L).

**FIGURE 3:**
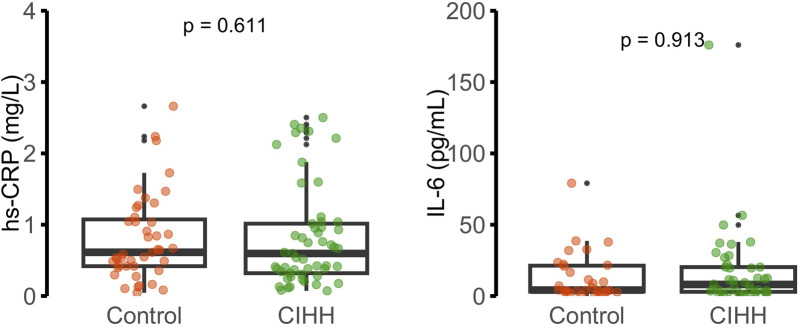
Inflammatory markers: hs-CRP (mg/L) and IL-6 (pg/mL).

**FIGURE 4:**
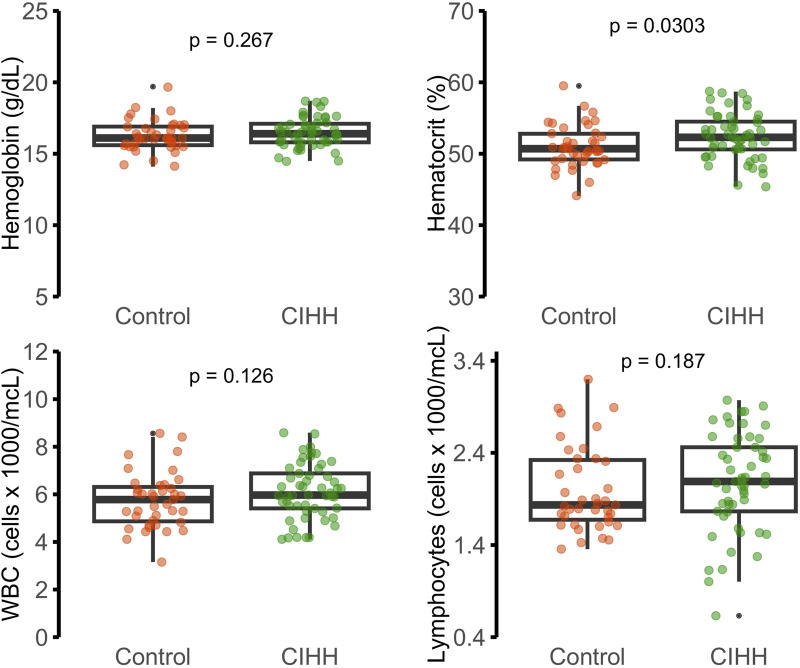
Hematological parameters: hemoglobin (g/dL), hematocrit (%), white blood cells (WBC, cells × 1,000/mcL), and lymphocytes (cells × 1,000/mcL).

Biochemical and hematological findings are presented in Figures 2–4. No statistically significant differences were observed between CIHH and control participants in total cholesterol, LDL-C, HDL-C, triglycerides, fasting glucose, or serum insulin (p > 0.05 for all). Similarly, inflammatory biomarkers, including hs-CRP and IL-6, did not differ significantly between groups (p = 0.611 and p = 0.913, respectively).

As expected, under intermittent hypobaric exposure, the median hematocrit value was significantly higher in the CIHH group (52.3%) than in the control group (50.7%) (p = 0.03). In contrast, no significant differences were observed in hemoglobin concentration, total white blood cell count, or lymphocyte count (p > 0.05). These results indicate a mild erythropoietic adaptation to hypoxia without evidence of systemic inflammatory activation.

**FIGURE 5:**
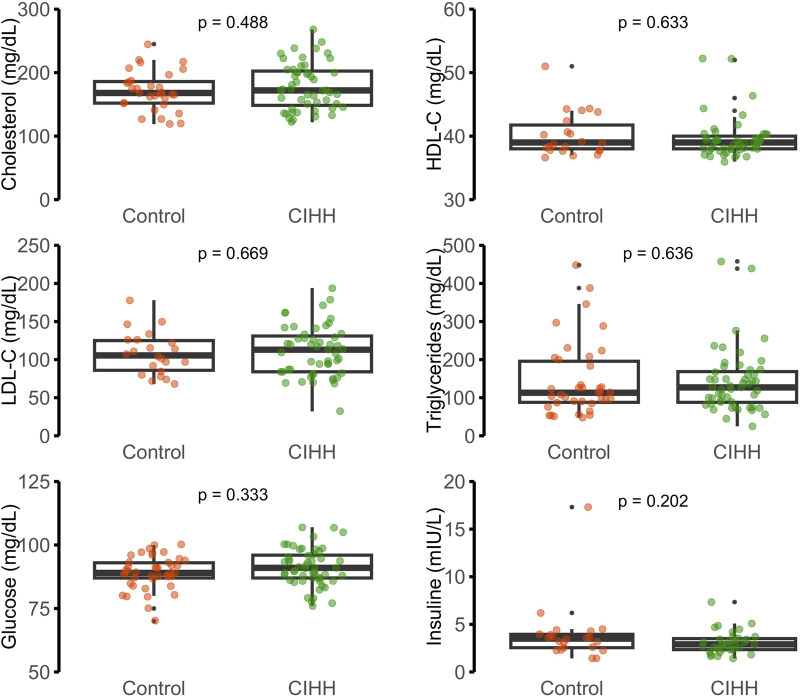
CVD risk in 10 years according to the Reynolds and Framingham scores (%).

Cardiovascular risk, estimated using the RRS and FRS (Figure 5), was slightly higher in the CIHH group (1.4% and 2.0%) than in controls (1.3% and 1.6%, respectively); however, these differences were not statistically significant (p > 0.05). Overall, both groups exhibited low predicted cardiovascular risk, consistent with a generally healthy working population, despite periodic hypoxic exposure.

Taken together, workers intermittently exposed to high altitude demonstrated metabolic, anthropometric, and inflammatory profiles comparable to those of individuals working permanently at low altitude. The only significant differences were higher hematocrit levels and a greater prevalence of obesity among CIHH workers. Within the exposure parameters assessed, chronic intermittent hypobaric hypoxia does not appear to exert clinically meaningful effects on cardiometabolic health.

## 4 Discussion

In this study, we characterize the cardiometabolic and hematological profile of male workers exposed to CIHH through rotational shifts at high altitude (3,000–4,500 m), interspersed with recovery periods at low altitude. Two findings stand out: first, CIHH exposure was associated with a modest but significant elevation in hematocrit, consistent with an erythropoietic adaptation to hypobaric hypoxia. Second, obesity prevalence—defined categorically using standard BMI thresholds—was higher among CIHH workers, whereas continuous anthropometric indices, body-composition measures, metabolic biomarkers, inflammatory markers, physical activity, obstructive sleep apnea risk, and estimated cardiovascular risk were broadly comparable between groups. Collectively, these data support a model of selective hematological adaptation in CIHH without demonstrable short-term perturbation of systemic cardiometabolic or inflammatory profiles within the exposure range and duration examined.

To ensure physiological and occupational consistency, altitude strata were defined according to established standards (Pollard and Murdoch, 2003; Savioli et al., 2022). The CIHH exposure profile in this cohort falls within the high-to-very-high altitude range, where hypobaric hypoxia is sufficient to elicit well-described oxygen-transport adaptations (Viscor et al., 2018; Brito et al., 2018; Richalet and Lhuissier, 2015). The higher hematocrit observed among CIHH workers aligns with a controlled erythropoietic response and does not, in this cohort, suggest maladaptive polycythemia, given the absence of chronic mountain sickness features (González-Candia et al., 2023; Esenamanova et al., 2014). Similar patterns have been documented in occupational high-altitude shift models, reinforcing the reproducibility of this hematological signature under intermittent exposure (Aragón-Vela et al., 2020).

The higher prevalence of obesity among CIHH workers merits particular caution in interpretation. The categorical difference in obesity occurred despite the absence of significant between-group differences in BMI treated continuously and in multiple continuous indicators of adiposity, including body fat percentage, visceral fat, waist-to-height ratio, and conicity index. This discordance increases the possibility that the apparent association is, at least partly, sensitive to dichotomization around predefined cutoffs rather than reflecting a consistent shift in the underlying distribution of adiposity. Methodological work has long emphasized that categorizing continuous variables can amplify apparent group differences and increase the risk of inferential overreach if the underlying distributions are not concurrently considered (Altman et al., 2006; Royston et al., 2006). Accordingly, the present findings should be framed as a descriptive epidemiological association within a specific occupational context, rather than as evidence that intermittent hypoxia directly increases adiposity.

In CIHH-exposed workforces, body weight and metabolic risk are shaped by a complex interplay of determinants that extend beyond hypoxic exposure alone. Rotational schedules frequently co-occur with circadian misalignment, altered sleep architecture, constrained food environments, and recovery periods at low altitude—all of which can influence energy balance and weight trajectories. Consistent with this, prior studies in mining and other CIHH contexts report heterogeneous associations between intermittent hypoxia, body mass, and metabolic risk, plausibly reflecting variation in work organization and behavioral ecology across settings (Woolcott et al., 2016; Esenamanova et al., 2014; Siques et al., 2020; Kayser and Verges, 2021). Notably, this phenotype differs from that of permanent high-altitude residents, who often exhibit lower body mass and adiposity—patterns more consistent with sustained exposure and chronic energy imbalance than with intermittent hypoxia *per se* (Cui et al., 2020; Woolcott et al., 2016).

Although experimental and clinical literature has proposed that intermittent hypoxia may influence metabolic regulation through adipose-tissue signaling, adipokine modulation, and hypoxia-responsive pathways, these mechanisms were not assessed in the present study. Consequently, mechanistic interpretations cannot be drawn from the current dataset and should be explicitly positioned as testable hypotheses for future work rather than explanatory conclusions (Drager et al., 2010; Mallet et al., 2018). Future longitudinal studies incorporating direct endocrine and molecular measurements will be required to delineate the boundary conditions under which CIHH might contribute to metabolic change.

Despite evidence linking prolonged CIHH exposure to vascular remodeling and endothelial dysfunction in specific long-term mining cohorts (Aragón-Vela et al., 2020; Lüneburg et al., 2017; Brito et al., 2007), we observed no between-group differences in estimated 10-year cardiovascular risk. Both Framingham and Reynolds scores remained low, which is unsurprising given the relatively young age and low baseline risk profile of the cohort. These findings align with population-based analyses, indicating that traditional risk scores may have limited discriminatory utility in younger adults and may be insufficiently sensitive to detect early risk divergence in such settings (Hernández-Vásquez et al., 2022; Cook et al., 2012).

Inflammatory markers (hs-CRP and IL-6) were also comparable between groups. Although acute hypoxic exposure can induce transient inflammatory activation (Turnbull, 2018; Hartm et al., 2000), repeated intermittent exposure may favor adaptive tolerance through antioxidant and anti-inflammatory responses, yielding a neutral systemic inflammatory profile under stable occupational patterns (Bonnitcha et al., 2018; Katuntsev et al., 2021; Gassmann and Muckenthaler, 2015). The absence of inflammatory divergence in the present cohort is therefore compatible with an interpretation of physiological accommodation rather than chronic systemic stress.

From an occupational health standpoint, the high prevalence of obesity observed among CIHH workers remains clinically salient irrespective of causality. Obesity has been associated with reduced work fitness and increased likelihood of early withdrawal from high-altitude employment (Vinnikov and Krasotski, 2022). In practice, preventive programs targeting nutrition, sleep–wake regularity, circadian alignment, and work–rest design may be the most tractable avenues to mitigate long-term cardiometabolic burden in CIHH-exposed populations.

In summary, CIHH in this occupational context is characterized by a reproducible hematological adaptation and otherwise broadly preserved cardiometabolic, inflammatory, and behavioral profiles in the short term. Future prospective studies—ideally incorporating quantitative hypoxic dose metrics, subclinical cardiovascular phenotyping, and endocrine/molecular measures—are needed to clarify long-term trajectories and to distinguish contextual drivers of obesity from any exposure-specific contributions.

To address the possibility that the obesity finding was driven by classification thresholds, we performed sensitivity analyses using continuous and alternative adiposity measures (Supplementary Material). These analyses suggest that the observed difference is sensitive to BMI categorization rather than indicative of a consistent physiological shift in adiposity. Accordingly, causal interpretations were further tempered.

## 5 Conclusion

In conclusion, male workers exposed to chronic intermittent hypobaric hypoxia through rotational high-altitude schedules demonstrated a consistent hematological adaptation, manifested as moderately higher hematocrit levels, without accompanying alterations in metabolic, inflammatory, behavioral, or estimated cardiovascular risk profiles. Within the exposure range and duration examined, CIHH did not appear to be associated with clinically meaningful disturbances in systemic cardiometabolic health.

The higher prevalence of obesity observed among CIHH workers, when defined using categorical BMI thresholds, occurred in the absence of parallel differences in continuous anthropometric or body-composition measures. This pattern suggests that the finding is best interpreted as a descriptive epidemiological characteristic of this occupational setting, potentially influenced by contextual and methodological factors, rather than as evidence of a direct physiological effect of intermittent hypoxia on adiposity.

From an occupational health perspective, these results highlight the importance of addressing modifiable contextual determinants—such as work organization, nutritional environment, sleep–wake regulation, and recovery patterns—to support long-term cardiometabolic well-being in rotational high-altitude workforces. Overall, CIHH in this cohort was associated with selective hematological accommodation, whereas broader cardiometabolic and inflammatory profiles remained largely preserved.

Future prospective investigations incorporating quantitative hypoxic dose metrics, longitudinal follow-up, and direct assessment of metabolic and endocrine pathways will be essential to clarify long-term health trajectories and to disentangle exposure-specific effects from occupational and environmental influences in intermittent high-altitude settings.

## 6 Limitations

This study has several limitations that should be considered when interpreting its findings. First, although the sample is representative of the occupational settings evaluated, the modest sample size may have limited statistical power to detect subtle differences in cardiometabolic and inflammatory outcomes characterized by substantial interindividual variability.

Second, the cross-sectional design captures a single time point within the adaptation process to intermittent hypoxia and therefore precludes causal inference. Although the present analysis provides a detailed descriptive profile of workers exposed to CIHH, longitudinal studies are required to determine temporal trajectories and long-term health implications.

Third, exposure to chronic intermittent hypobaric hypoxia was operationally defined using altitude ranges and rotational work schedules. Precise quantification of cumulative hypoxic dose—integrating altitude intensity, duration of exposure, frequency of cycles, and total cumulative time—was beyond the scope of this study. The absence of a quantitative hypoxic dose metric limits the ability to explore dose–response relationships and to assess biological plausibility with greater resolution.

Fourth, with the exception of obesity, most outcomes were evaluated using unadjusted between-group comparisons. Although this approach is appropriate for exploratory and descriptive analyses, residual confounding by factors such as age, birthplace altitude, behavioral characteristics, work-rotation patterns, and years of exposure cannot be excluded. Accordingly, findings for these outcomes should be interpreted as descriptive rather than causal.

Finally, cardiovascular risk was estimated using the Framingham and Reynolds risk scores. These instruments have limited discriminative performance in relatively young male populations with low baseline cardiovascular risk. Consequently, the absence of differences in estimated risk between groups may reflect methodological limitations of the risk scores rather than true equivalence in long-term cardiovascular risk. Future studies incorporating alternative risk stratification tools and subclinical cardiovascular markers may provide greater sensitivity in this context.

## Data Availability

The datasets generated and analyzed during the present study are not publicly available due to institutional policies on participant confidentiality but are available from the corresponding author upon reasonable request.
