# Supplementary material for: Cardiovascular risk and obesity in miner workers exposed to intermittent hypobaric hypoxia in the Peruvian Andes
Source: Front Physiol. 2026 Mar 10;17:1693470. doi: 10.3389/fphys.2026.1693470 (PMC13011168; doi:10.3389/fphys.2026.1693470)
Supplement: Supplementary file 1 [file Presentation1.pdf]

Supplementary Figures

Supplementary Figure S1.

Distribution of body mass index (BMI) as a continuous variable in CIHH workers and low-altitude controls.

Individual-level BMI values are shown with group-level summary statistics. Distributions largely overlap between groups, indicating no consistent shift in continuous adiposity despite differences observed when BMI is categorized using standard cut-offs.

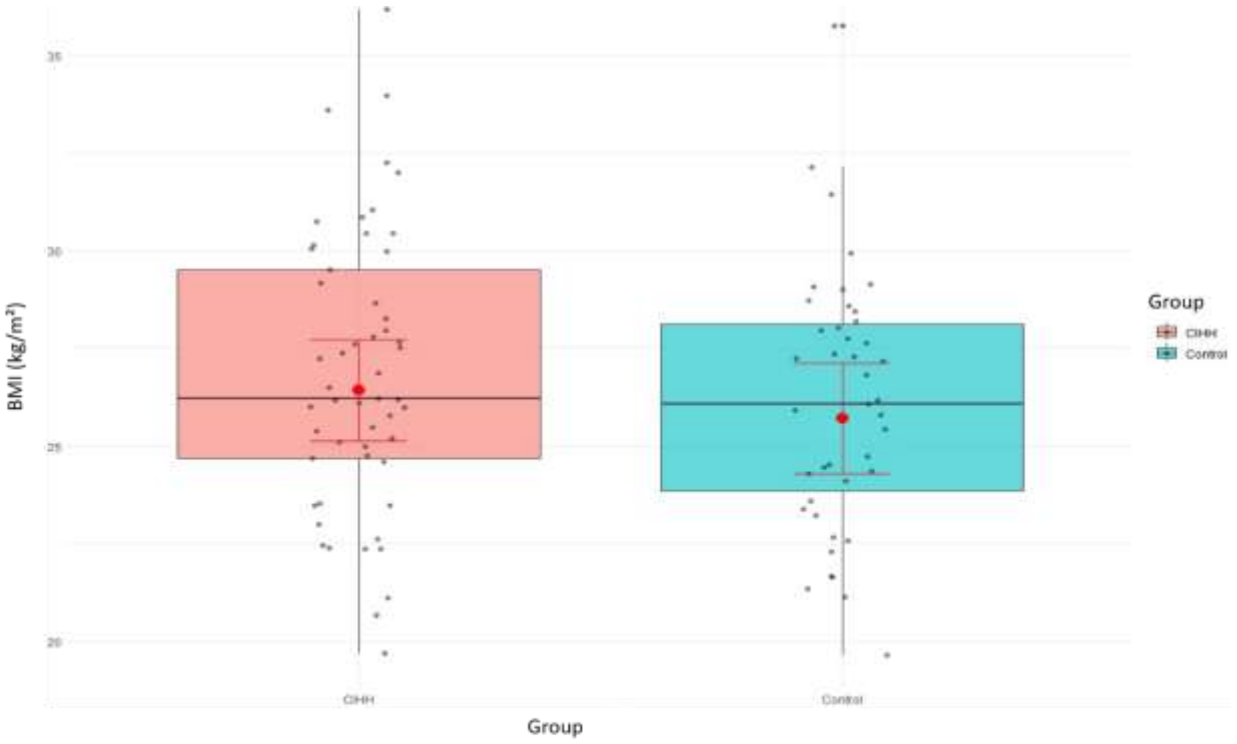

Supplementary Figure S2

Prevalence of overweight and obesity (BMI ≥25 kg/m²) in CIHH workers and controls.

The between-group difference emerges only when BMI is dichotomized, highlighting the sensitivity of obesity prevalence estimates to classification thresholds.

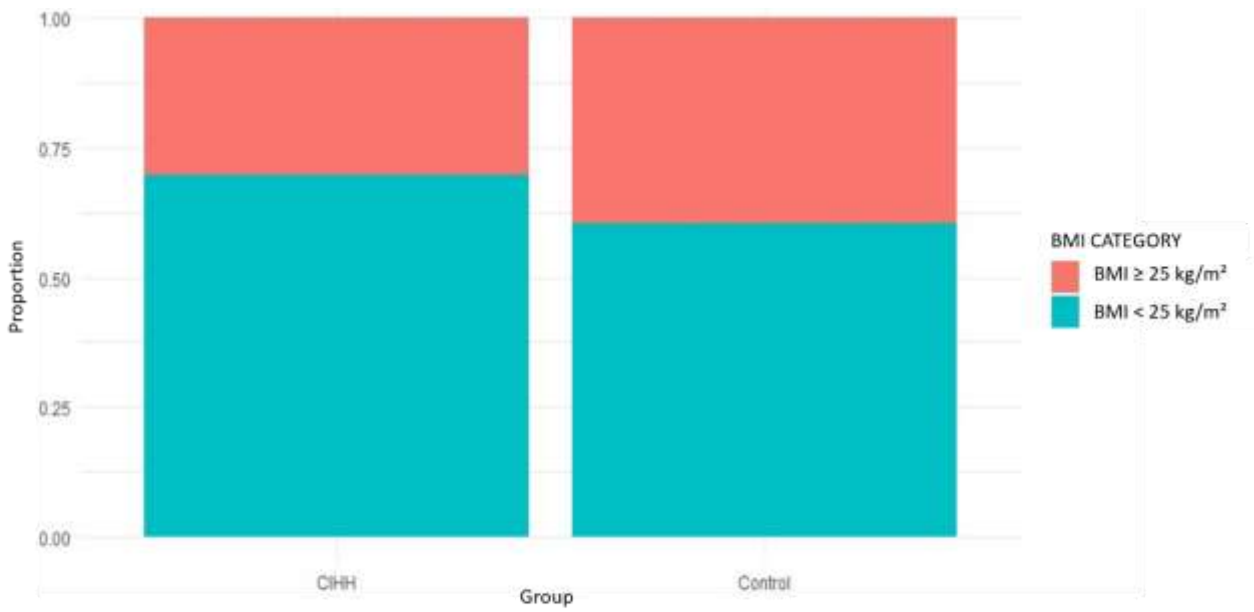

Supplementary Figure S3

Body fat percentage by group (CIHH vs control).

Direct measures of adiposity show overlapping distributions between groups, suggesting no robust difference in total body fat accumulation associated with CIHH exposure.

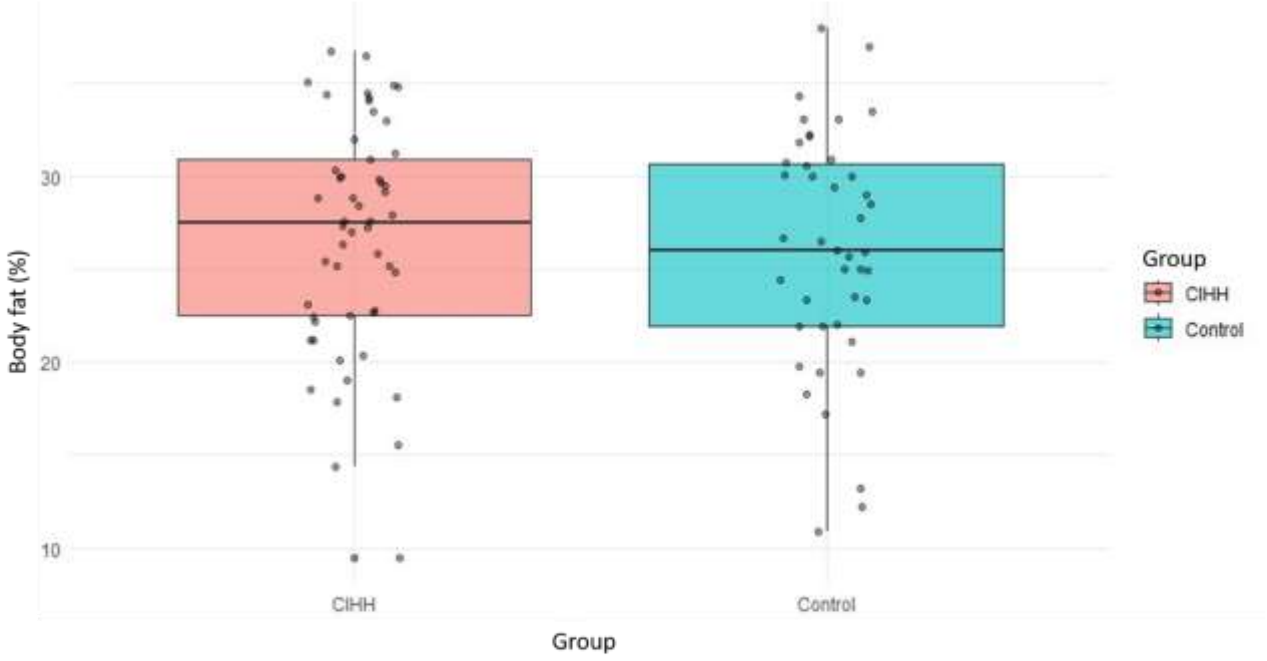

Supplementary Figure S4

Waist-to-height ratio (WHtR) by group.

Central adiposity assessed as a continuous variable demonstrates substantial overlap between CIHH workers and controls, supporting the absence of a consistent central fat redistribution.

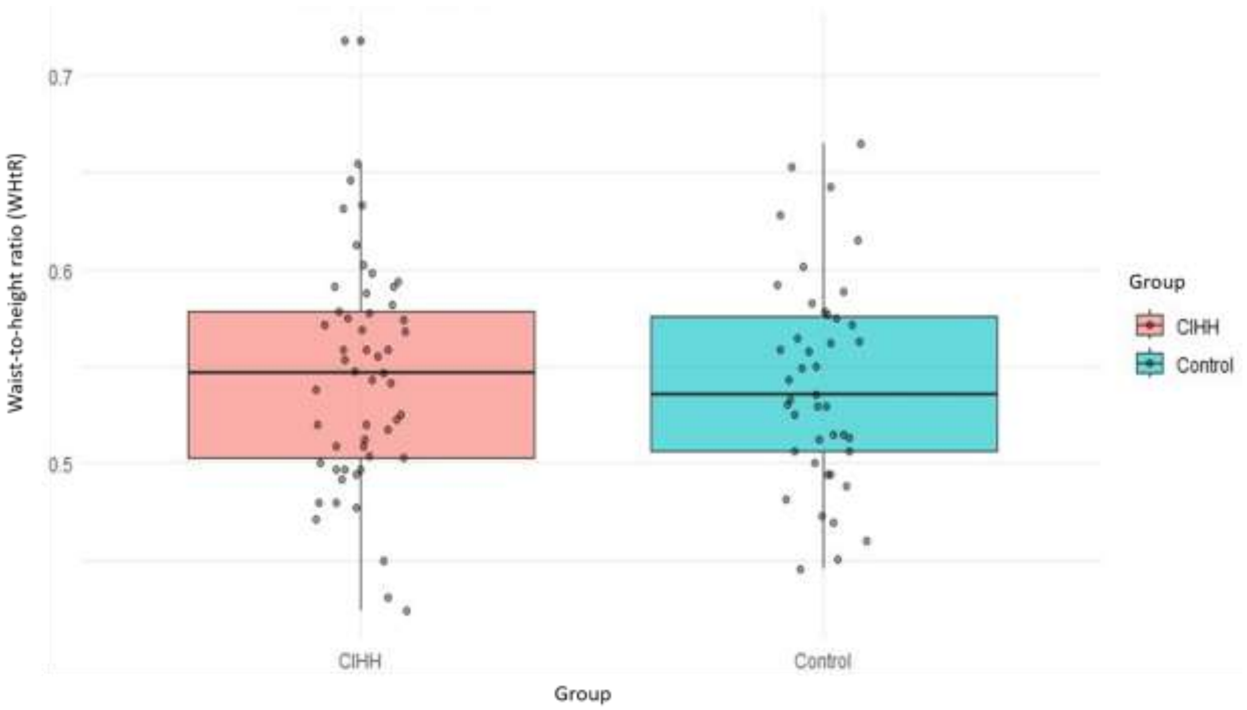

Supplementary Figure S5

LDL cholesterol distribution and prevalence of dyslipidemia (LDL ≥130 mg/dL) by group.

Individual LDL levels and categorical dyslipidemia prevalence are shown to complement cardiovascular risk score analyses in this young male cohort.

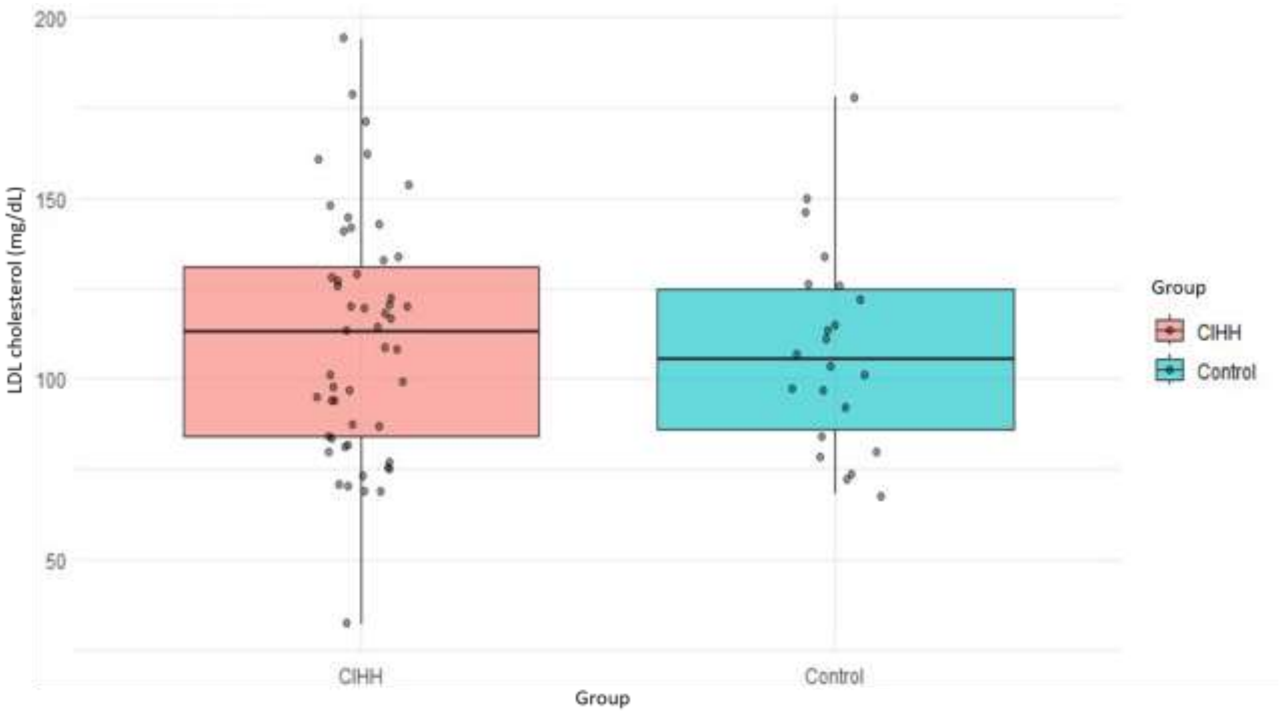

Supplementary Table

Table S1. Multivariable-adjusted association between CIHH exposure and cardiometabolic outcomes

Outcome	β (Control vs CIHH)	95% CI	p-value
BMI (kg/m²)	−0.71	−2.43 to 1.01	0.419
Body fat (%)	0.43	−2.77 to 3.64	0.791
Waist-to-height ratio (WHtR)	−0.003	−0.028 to 0.022	0.787
HDL cholesterol (mg/dL)	0.51	−1.41 to 2.42	0.607
LDL cholesterol (mg/dL)	10.30	−1.23 to 21.82	0.247
Fasting glucose (mg/dL)	−1.38	−4.60 to 1.85	0.491
hsCRP (log-transformed)	0.14	−0.32 to 0.60	0.554

Abbreviations: CIHH, chronic intermittent hypobaric hypoxia; BMI, body mass index; WHtR, waist-to-height ratio; HDL, high-density lipoprotein; LDL, low-density lipoprotein; hsCRP, high-sensitivity C-reactive protein.
Interpretation: Across outcomes, the adjusted group effect was small and not statistically

significant, indicating broadly comparable cardiometabolic profiles between CIHH workers and low-altitude controls after accounting for demographic and exposure-related covariates.

Supplementary Note

Note S1. Proxy of hypoxic dose

A direct physiological quantification of cumulative hypoxic dose (e.g., arterial oxygen saturation profiles or hypoxic load indices) was not available in the present study. To improve exposure characterization beyond rotation schedules and broad altitude categories, we derived a pragmatic proxy of hypoxic exposure based on the information available in the dataset.

Specifically, cumulative exposure was approximated using years of exposure, combined with the documented work rotations and time spent at altitudes above 3000 m. This proxy does not capture short-term physiological variability but provides a quantitative representation of long-term, repeated exposure to hypobaric hypoxia, which is consistent with the operational definition of chronic intermittent hypobaric hypoxia (CIHH) in occupational settings.

This exposure proxy was included as an adjustment variable in multivariable analyses (Supplementary Table S1) to account for inter-individual differences in cumulative hypoxic exposure. Results should be interpreted with caution, as this metric represents an approximation rather than a direct physiological measure; however, it allows partial quantification of hypoxic dose and strengthens the analytical framework relative to unquantified exposure descriptions.
